# Two decades of immunogenic cell death research: a bibliometric analysis of the 100 most cited publications

**DOI:** 10.3389/fonc.2026.1904609

**Published:** 2026-07-22

**Authors:** Li Guan, Lijuan Guo, Yuqiu Liu, Shengwei Bao, Mingyang Chen, Jia Luo, Huan Zhang, Sen Yang

**Affiliations:** 1 Department of Oral and Maxillofacial Surgery, Suining Central Hospital, Suining, China; 2 Department of Medical Cosmetology, Suining Central Hospital, Suining, China; 3 School of Stomatology, Zunyi Medical University, Zunyi, China

**Keywords:** bibliometric analysis, calreticulin exposure, immunogenic cell death, research trends, tumor immunology

## Abstract

**Background:**

Immunogenic cell death (ICD) represents a pivotal concept linking tumor cell demise with the activation of adaptive antitumor immunity. Since the concept was formalized around 2005, ICD has substantially reshaped research in tumor immunology and cancer therapy. However, bibliometric mapping and structured interpretation of the influential literature that has shaped this field remain limited.

**Methods:**

This bibliometric analysis, supplemented by structured narrative interpretation, used the Web of Science Core Collection and Scopus to identify the 100 most-cited ICD-related publications. Publication characteristics, citation patterns, journals, authors, institutions, countries/regions, and keywords were extracted and standardized. VOSviewer and CiteSpace were employed to construct collaboration networks, perform keyword co-occurrence and clustering analyses, and identify research hotspots and thematic evolution over time.

**Results:**

The top 100 most-cited ICD publications were published between 2005 and 2023. Within this highly cited publication set, more included papers were published after 2011, and the largest number of top-cited publications appeared in 2019. Highly cited studies were dominated by contributions from the United States and Western Europe, particularly France, with strong international collaboration networks. A small core of authors and institutions accounted for a substantial proportion of influential output. Keyword and burst analyses revealed a clear evolution from early work defining ICD hallmarks and antigen presentation mechanisms toward later integration with immunotherapy, nanomedicine, phototherapy, and engineered delivery platforms.

**Conclusion:**

Over the past two decades, ICD research has evolved from conceptual and mechanistic foundations toward a multidisciplinary framework with increasing relevance to tumor immunology, immunotherapy, and combination cancer therapy. Future progress will depend on function-based validation of immunogenicity, microenvironment-aware biomarkers, and rational therapeutic combinations to enhance clinical translatability.

## 1 Introduction

For decades, cell death was largely regarded as a passive biological endpoint with limited immunological relevance (1). This view has been reshaped over the last twenty years. Around 2005, Kroemer and colleagues introduced the concept of immunogenic cell death (ICD) to describe forms of cell death capable of activating the host immune system and eliciting antigen-specific immune responses against antigens released or exposed by dying cells (2). This paradigm provided a new theoretical foundation for tumor immunology and therapeutic strategies, marking a new phase in the study of cell death.

A defining feature of ICD is the release or surface exposure of a series of damage-associated molecular patterns (DAMPs) by dying cells (3–5), including ecto-calreticulin exposure, active ATP secretion, and the release of high-mobility group box 1 (HMGB1) (6–8). These signals can be sensed by antigen presenting cells (APC) such as dendritic cells, thereby promoting the uptake, processing, and presentation of tumor antigens and ultimately initiating adaptive immune responses (9, 10). Unlike conventional descriptions of apoptosis and necrosis, ICD emphasizes that the death process itself conveys immunological instructions rather than serving merely as a tissue clearing event (11).

Following its introduction, ICD rapidly became closely intertwined with cancer therapy research. Several conventional chemotherapeutics (12, 13), radiotherapy (14), and photodynamic therapy (15) have been shown to induce ICD under specific conditions, enhancing antitumor immunity while directly killing tumor cells. More recently, ICD has been recognized as an important determinant of responses to immune checkpoint inhibitors, further elevating its role in multimodal cancer treatment (16, 17).

Over the past two decades, publications on ICD have grown rapidly, and the scope has expanded from early conceptual validation to molecular mechanisms (18), induction strategies (19), biomarkers (20, 21), and translational applications (22). However, the evolution of the field has not been strictly linear; different stages have featured distinct focal topics, enabling technologies, and landmark papers. The landmark studies and thematic shifts that shaped ICD research have not been comprehensively mapped using bibliometric methods.

Bibliometric analysis offers an objective approach to mapping disciplinary development (23). By systematically interrogating highly cited papers, one can identify the most influential findings, leading authors and institutions, and track the evolution of research themes (24). Given that nearly two decades have elapsed since the concept of ICD was articulated, a systematic analysis of the 100 most cited ICD-related publications can help summarize the field’s knowledge base and development trajectory, providing a reference for future basic and translational work. Accordingly, taking “two decades of immunogenic cell death” as the temporal backdrop, this study performs bibliometric and content analyses of the 100 most cited papers in the field to elucidate research hotspots, developmental stages, and potential future directions.

## 2 Methods

### 2.1 Data sources and search strategy

A bibliometric search was performed in the Web of Science Core Collection (WoSCC) and Scopus on 12 December 2025. No publication-year restriction was applied. The search terms were “immunogenic cell death” and “cell death, immunogenic”. In WoSCC, the search formula was TS = (“immunogenic cell death” OR “cell death, immunogenic”). In Scopus, the formula was TITLE-ABS-KEY (“immunogenic cell death” OR “cell death, immunogenic”). Full bibliographic records and citation information were exported for further analysis. Because the aim of this study was to map the highly cited literature explicitly centered on ICD, we adopted a high-specificity search strategy using the established ICD terminology rather than a broad search of all DAMP-, tumor immunity-, or cancer therapy-related publications.

### 2.2 Study selection

Records retrieved from WoSCC and Scopus were first ranked according to citation counts within each database. Because the objective of this study was to identify the 100 most-cited ICD-centered publications rather than to review all ICD-related publications, we used an *a priori* oversampling strategy by exporting the top 200 most-cited records from each database, corresponding to twice the final target sample size. The two datasets were merged, and duplicate records were removed based on DOI, title, first author, journal, and publication year. To evaluate whether the top-200 export window was sufficient, we performed a rank-threshold sensitivity analysis. After duplicate removal and manual relevance screening, the exported records contained enough eligible ICD-centered publications to identify the final top 100. The 100th-ranked included publication had 257 WoSCC citations. The exported top-200 windows from the two databases provided a two-fold oversampling frame and contained sufficient eligible ICD-centered publications to construct the final top-100 dataset. This rank-threshold check supported the adequacy of the export strategy for the present top-cited analysis.

The remaining publications were screened manually for relevance to immunogenic cell death (ICD). Eligible publications were original articles or reviews in which immunogenic cell death was a central topic, mechanism, endpoint, or interpretive framework. Studies addressing ICD-related DAMPs, tumor immunity, cancer therapy, nanomedicine, phototherapy, radiotherapy, chemotherapy, or immune checkpoint blockade were included only when they explicitly linked these topics to ICD. Publications that discussed DAMPs, tumor immunity, or cancer therapy in a general manner without a clear ICD focus were excluded as marginally relevant. Publications with only marginal relevance to ICD, as well as editorials, letters, corrections, meeting abstracts, news items, and non-English publications, were excluded. The eligible publications were subsequently ranked according to WoSCC citation counts, and the top 100 most cited ICD-related publications were included in the final analysis. The complete list of the 100 included publications, including title, publication year, journal, DOI, WoSCC citation count, Scopus citation count, and article type, is provided in **Supplementary Table 1**.

### 2.3 Data extraction and normalization

The following fields were extracted from the included publications: publication year, journal, authors, author affiliations, country/region, institution, keywords, and citation counts (WoS/Scopus). Manual normalization was performed before network construction. Author names were standardized by unifying initials, spelling variants, and name-order differences. Institutional names were harmonized by merging abbreviations, English variants, and affiliated subunits when they clearly referred to the same parent institution. Country/region names were standardized according to the affiliation information provided in the bibliographic records. Keyword normalization included merging abbreviations with full terms, singular and plural forms, hyphenated and non-hyphenated variants, and closely synonymous terms (**Supplementary Table 2**). Terms were not merged when they represented distinct biological mechanisms, therapeutic modalities, or analytical concepts. All uncertain cases were discussed by two authors until consensus was reached. Country/region and institution analyses were performed using full counting based on all author affiliations; therefore, the summed counts across countries/regions or institutions could exceed the total number of included publications. These rankings should be interpreted as indicators of affiliation-based participation and collaborative visibility within the top-cited ICD publication set, rather than as independent or fractionalized measures of institutional or national impact.

### 2.4 Bibliometric indicators and visualization analyses

The annual distribution of the top 100 most-cited publications was calculated to describe the publication-year pattern within the highly cited ICD literature set. This analysis was not intended to represent total annual productivity of the ICD field. Journal distribution and outputs by countries/regions and institutions were calculated to characterize the contribution landscape of the included highly cited publications.

Bibliometric analyses were conducted using VOSviewer version 1.6.20, CiteSpace version 6.4.R1, R version 4.5.0, and the bibliometrix R package version 4.2.3. Full records and citation data were exported from WoSCC and Scopus in plain text/CSV format. After duplicate removal and manual relevance screening, bibliographic information from the final 100 publications was imported into VOSviewer, CiteSpace, and bibliometrix for quantitative and visualization analyses.

For co-authorship and collaboration analyses, authors, institutions, and countries/regions were analyzed using full counting. Because the final dataset contained only 100 publications, low inclusion thresholds were used to preserve the structure of the highly cited literature network. Unless otherwise specified, the minimum threshold was set to 1 publication for author, institution, and country/region analyses, and 1 occurrence for keyword analyses. Network clustering in VOSviewer was performed using association-strength normalization and the built-in VOSviewer clustering algorithm, with the resolution parameter set at 1.00. Node size represents publication count or keyword frequency, and link strength represents co-authorship, collaboration, or co-occurrence intensity (25). Keyword analyses were based on author keywords and database-indexed keywords available in the exported WoSCC and Scopus records. Before analysis, keywords were manually standardized by merging singular and plural forms, abbreviations and full names, spelling variants, and synonymous terms. No additional pruning was applied in VOSviewer.

CiteSpace was used for keyword timeline visualization and burst detection (26). The time slicing was set from 2005 to 2023, with one year per slice. The node type was set as Keyword. The selection criterion was set as g-index, k = 25. Pruning settings were retained as the software default unless otherwise specified. Burst detection was conducted using Kleinberg’s algorithm with default CiteSpace parameters, and the top 25 burst keywords were visualized (27).

### 2.5 Journal impact factor and geographic distribution analysis

Journal impact factor (IF) values were obtained from the 2024 Journal Citation Reports (JCR) and used as an indicator of journal academic influence. In addition, the bibliometrix package (v4.2.3) in R was used to analyze the geographic distribution of the included publications and to generate a world map of major countries and regions contributing to ICD research, thereby illustrating global academic participation and collaboration.

## 3 Results

### 3.1 Overview of the top 100 most-cited articles

**Figure 1:**
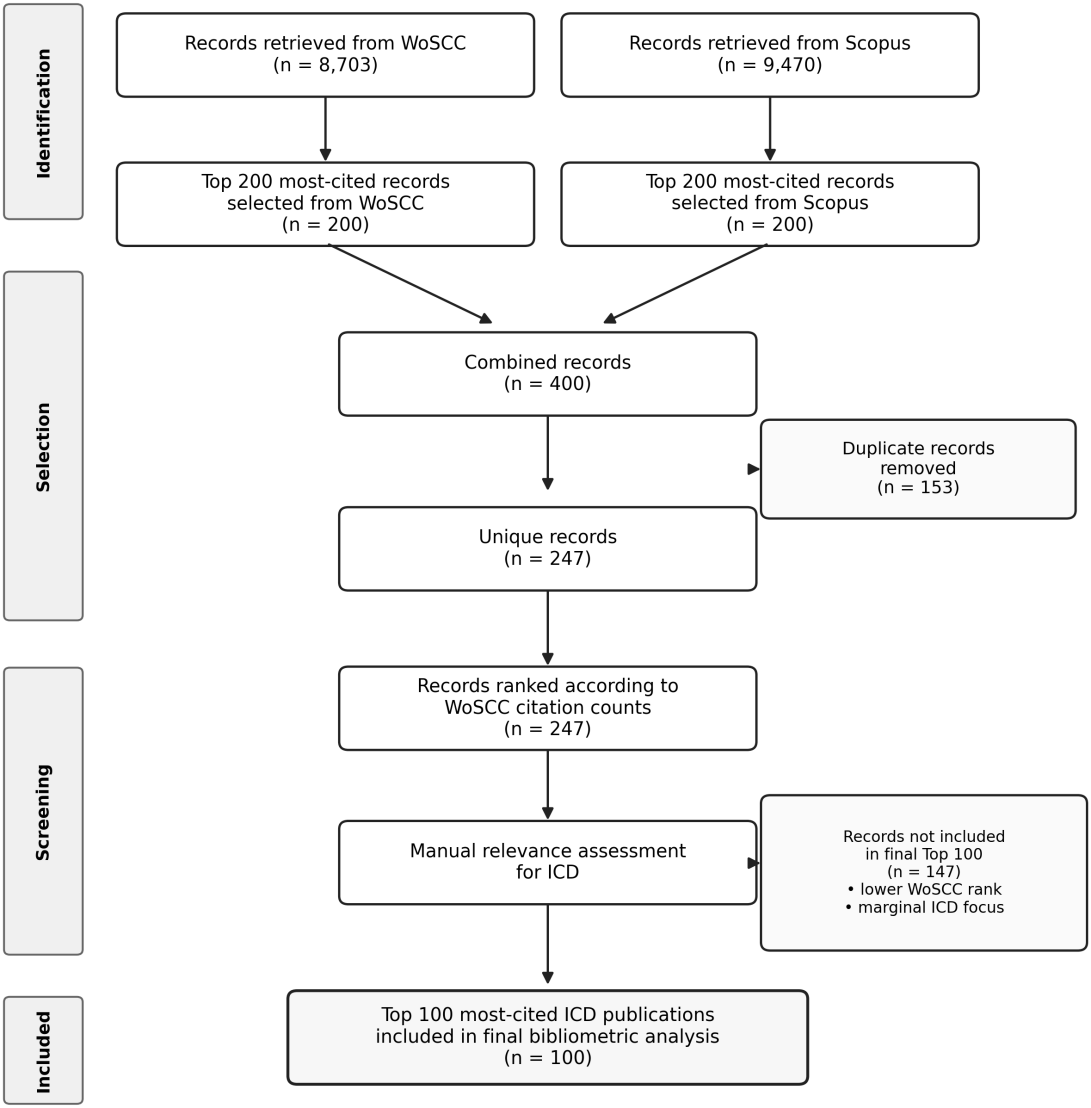
Literature retrieval and selection workflow.

**Figure 2:**
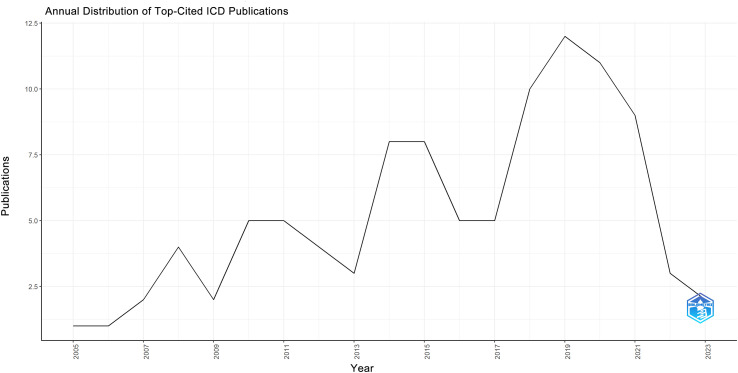
Publication-year distribution of the top 100 most-cited publications on ICD.

On 12 December 2025, 8,703 records were retrieved from the Web of Science Core Collection (WoSCC) and 9,470 records were retrieved from Scopus. To identify highly influential publications, the top 200 most cited records from each database were exported and merged. After duplicate removal, 247 unique publications remained. Based on the citation-ranked design, the top-200 window provided a two-fold oversampling frame for the intended top-100 analysis and contained sufficient eligible records to construct the final dataset. These publications were ranked according to WoSCC citation counts and manually assessed for relevance to immunogenic cell death (ICD), resulting in the inclusion of the 100 most cited ICD-related publications in the final analysis (**Figure 1**). Detailed bibliographic information for all 100 included publications is provided in **Supplementary Table 1**. The included publications were published between 2005 and 2023. As shown in **Figure 2**, the annual number of top-cited ICD publications increased gradually after the initial appearance of the field and increased more sharply after 2011. The highest number of top-cited publications appeared in 2019 (n = 12), followed by 2020 (n = 11). Among the 100 publications, 55 were original research articles and 45 were reviews. These papers accumulated 56,952 citations in WoSCC. Individual WoSCC citation counts ranged from 257 to 2,699, with a median of 394. In total, 631 authors from 27 countries/regions and 241 institutions contributed to these publications, which were distributed across 54 journals.

**Table 1** presents the top 10 most cited articles in the field of ICD. The leading paper, “Immunogenic cell death in cancer therapy (28),” published in 2013 in Annual Review of Immunology, ranked first with 2,699 citations in WoS and 2,813 citations in Scopus. The second ranked article, “Calreticulin exposure dictates the immunogenicity of cancer cell death (29),” published in 2007 in Nature Medicine, had 2,650 (WoS) and 2,756 (Scopus) citations, reflecting the field-defining role of calreticulin exposure as an immunogenic signal. The third ranked paper, “Immunogenic cell death and DAMPs in cancer therapy (30)” (Nature Reviews Cancer, 2012), accumulated 2,557 (WoS) and 2,623 (Scopus) citations, underscoring the centrality of DAMPs in conceptualizing ICD.

**Table 1:** Top 10 most cited publications on immunogenic cell death.

Rank	Article title	Source title	Publication year	IF (2024)	TC (WoS)	TC (scopus)
1	Immunogenic cell death in cancer therapy	Annual Review of Immunology	2013	33.3	2699	2813
2	Calreticulin exposure dictates the immunogenicity of cancer cell death	Nature Medicine	2007	50	2650	2756
3	Immunogenic cell death and DAMPs in cancer therapy	Nature Reviews Cancer	2012	66.8	2557	2623
4	Approaches to treat immune hot, altered and cold tumours with combination immunotherapies	Nature Reviews Drug Discovery	2019	101.8	2517	2607
5	Immunogenic cell death in cancer and infectious disease	Nature Reviews Immunology	2017	60.9	2375	2460
6	Caspase dependent immunogenicity of doxorubicin induced tumor cell death	Journal of Experimental Medicine	2005	10.6	1273	1321
7	Autophagy dependent anticancer immune responses induced by chemotherapeutic agents in mice	Science	2011	45.8	1126	1194
8	Immunostimulation with chemotherapy in the era of immune checkpoint inhibitors	Nature Reviews Clinical Oncology	2020	83.2	1030	1064
9	Immunogenic cell stress and death	Nature Immunology	2022	27.6	985	1009
10	Immunogenic death of colon cancer cells treated with oxaliplatin	Oncogene	2010	7.3	982	1012

IF, impact factor; TC, total citations.

### 3.2 Country/region contributions

**Figure 3:**
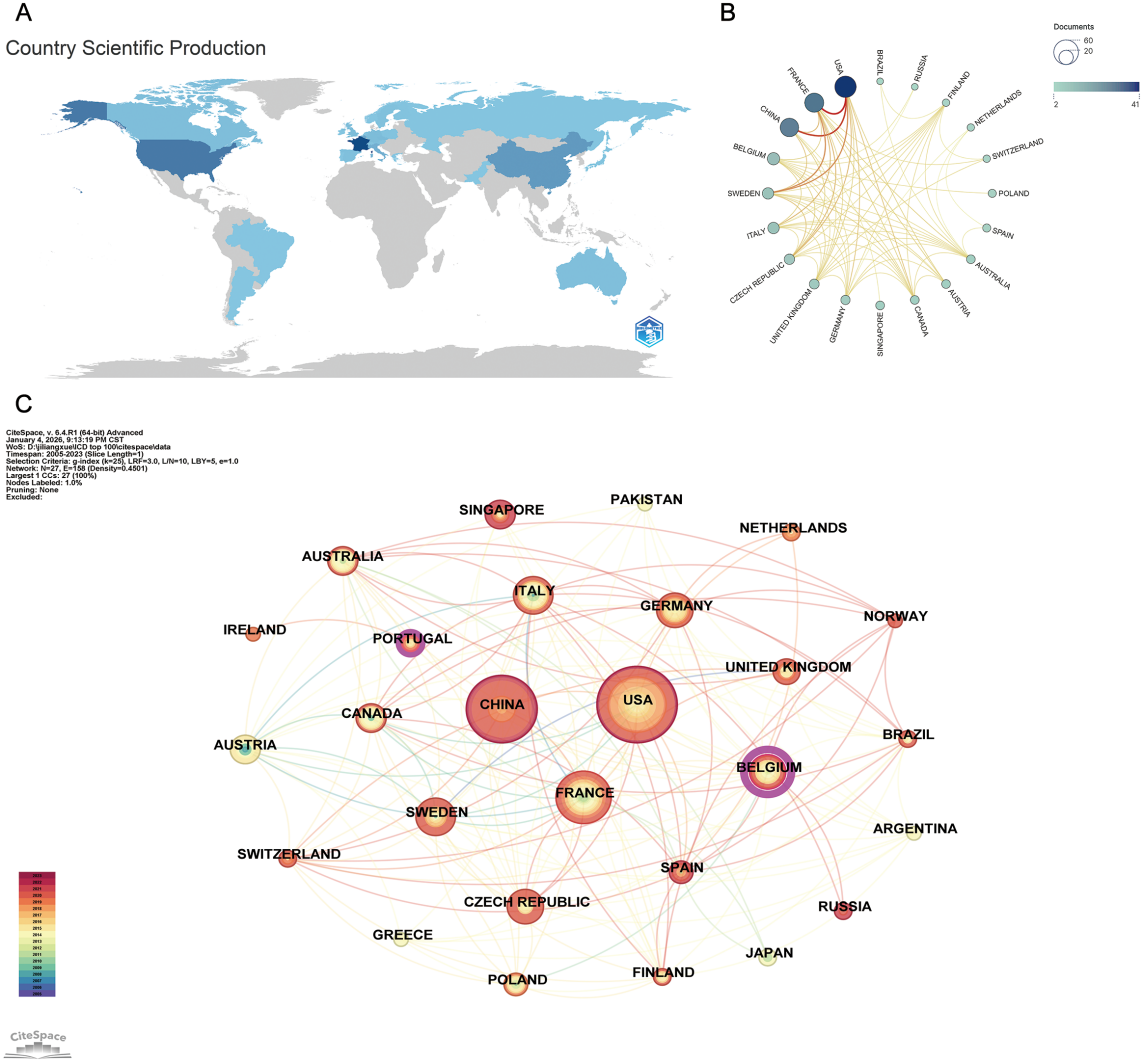
Cooperation between countries/regions. **(A)** Geographic distribution of publications by country/region. **(B)** Country/region collaboration network based on co-authorship. **(C)** Country/region collaboration map highlighting major cooperative links.

The geographic distribution of the included publications is shown in **Figure 3A**, and the leading contributing countries/regions are summarized in **Table 2**. The United States contributed the largest number of top-cited ICD publications (n = 41), followed by France (n = 32) and China (n = 30). France had the highest total citation count, with 26,106 WoSCC citations and 27,181 Scopus citations, exceeding the United States, which had 22,812 WoSCC citations and 23,910 Scopus citations. China ranked third in both publication count and citation totals, with 13,629 WoSCC citations and 14,155 Scopus citations. Italy and Sweden each contributed 10 publications, indicating notable representation among European countries.

**Table 2:** Top 6 countries/regions of top 100 most cited articles on immunogenic cell death.

Rank	Country/region	Count	Centrality	TC (WoS)	TC (scopus)
1	USA	41	0.03	22812	23910
2	FRANCE	32	0.06	26106	27181
3	CHINA	30	0.05	13629	14155
4	BELGIUM	12	0.48	7756	7854
5	ITALY	10	0.06	7679	8029
6	SWEDEN	10	0.03	7682	8011

Country/region collaboration networks are shown in **Figures 3B, C**. Belgium contributed fewer publications than the United States, France, and China, but it showed the highest betweenness centrality among the leading countries/regions (centrality = 0.48). This suggests that Belgium may have played a bridging role in international collaboration networks. By contrast, the United States, France, and China showed relatively high output and citation influence, whereas Belgium was more prominent in network connectivity. Because full counting was used, country/region counts represent affiliation-based participation in the included top-cited publications. Collaborative papers involving multiple countries/regions may therefore contribute to the counts of each participating country/region.

### 3.3 Distribution of research institutions

**Figure 4:**
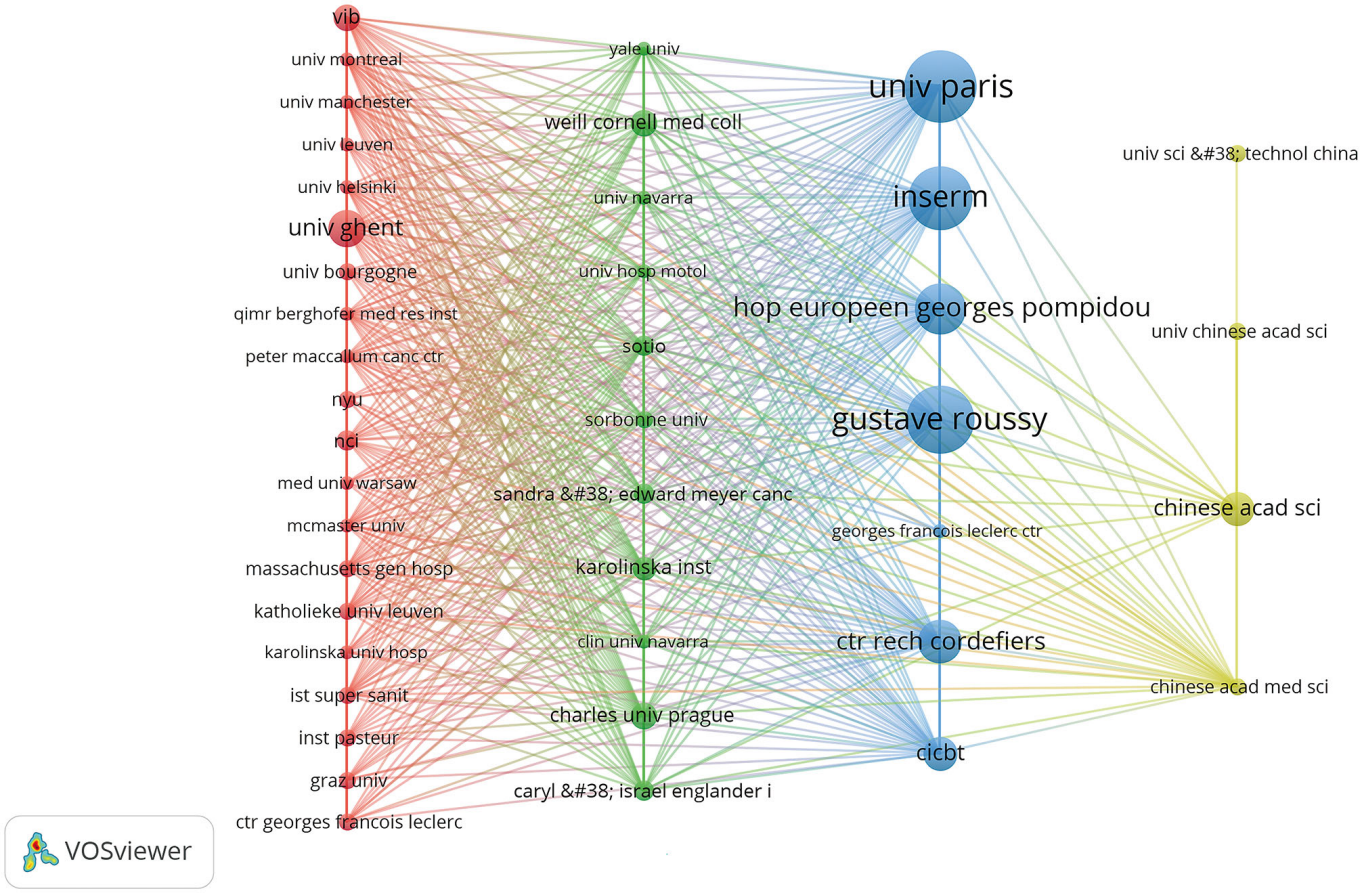
Institutions of the top 100 most cited articles on ICD.

Institution-level analysis showed that highly cited ICD research was concentrated in a limited number of leading institutions, particularly in France (**Figure 4**, **Table 3**). Université Paris ranked first with 27 top-cited publications and 23,417 WoSCC citations, followed by Gustave Roussy with 25 publications and 20,794 WoSCC citations, and INSERM with 23 publications and 20,180 WoSCC citations. Hôpital Européen Georges Pompidou and Centre de Recherche des Cordeliers also ranked among the leading institutions, further indicating the strong contribution of French research networks to the early and highly cited ICD literature.

**Table 3:** Top 10 institutions of the top 100 most cited articles on immunogenic cell death.

Institutions	Count	Centrality	TC (WoS)	Country
Univ Paris	27	0.04	23417	FRANCE
Gustave Roussy	25	0.05	20794	FRANCE
INSERM	23	0.18	20180	FRANCE
Hop Europeen Georges Pompidou	17	0.01	13841	FRANCE
Ctr Rech Cordeliers	14	0.12	11736	FRANCE
Univ Ghent	11	0.11	7440	BELGIUM
CICBT	10	0.34	8561	FRANCE
Chinese Acad Sci	10	0.15	3673	CHINA
Weill Cornell Med Coll	7	0	6644	USA
Charles Univ Prague	7	0.09	3704	CZECH REPUBLIC

Outside France, Ghent University contributed 11 publications with 7,440 WoSCC citations, while the Chinese Academy of Sciences contributed 10 publications with 3,673 WoSCC citations. Centrality analysis showed that CICBT had the highest institutional betweenness centrality (0.34), followed by INSERM (0.18), the Chinese Academy of Sciences (0.15), and Centre de Recherche des Cordeliers (0.12). These findings indicate that several institutions acted as important collaborative nodes in the highly cited ICD literature. However, because institution-level analysis was based on author affiliations using full counting, some leading French institutions may partly represent overlapping affiliations within the same collaborative networks rather than fully independent institutional contributions. Therefore, institutional rankings should be interpreted as indicators of affiliation-based knowledge production and collaboration structure.

### 3.4 Journal influence

The top 100 publications were distributed across multiple high-impact journals, reflecting both the biomedical core of ICD research and its expanding interdisciplinary interfaces (**Table 4**). Nature Communications published the greatest number of top 100 papers (n = 6) and accumulated 3,118 WoS citations and 3,161 Scopus citations (IF = 15.7; JCR Q1). Advanced Materials and Cell Death & Differentiation each published five Top 100 papers, with Advanced Materials exhibiting a high journal impact factor (IF 26.8; Q1), highlighting the increasing integration of ICD with materials science and nano-enabled therapeutic platforms. ACS Nano, Journal of the American Chemical Society, and Oncogene each contributed four top 100 papers, indicating that ICD-relevant breakthroughs are disseminated through both oncology journals and materials journals.

**Table 4:** Top 10 journals with the most cited articles on immunogenic cell death.

Rank	Source	Documents	TC (WoS)	TC (scopus)	IF (2024)	JCR (2024)
1	Nature Communications	6	3118	3161	15.7	Q1
2	Advanced Materials	5	2106	2155	26.8	Q1
3	Cell Death & Differentiation	5	2172	2296	15.7	Q1
4	ACS Nano	4	1197	1315	16.1	Q1
5	Journal of the American Chemical Society	4	1854	1899	15.7	Q1
6	Oncogene	4	2154	2241	7.3	Q1
7	Nature Reviews Immunology	3	3681	3839	60.9	Q1
8	Journal For Immunotherapy of Cancer	3	1691	1517	10.6	Q1
9	Nature Reviews Clinical Oncology	3	1684	1769	83.2	Q1
10	Science Translational Medicine	3	1449	1529	14.7	Q1

### 3.5 Analysis of authors

**Figure 5:**
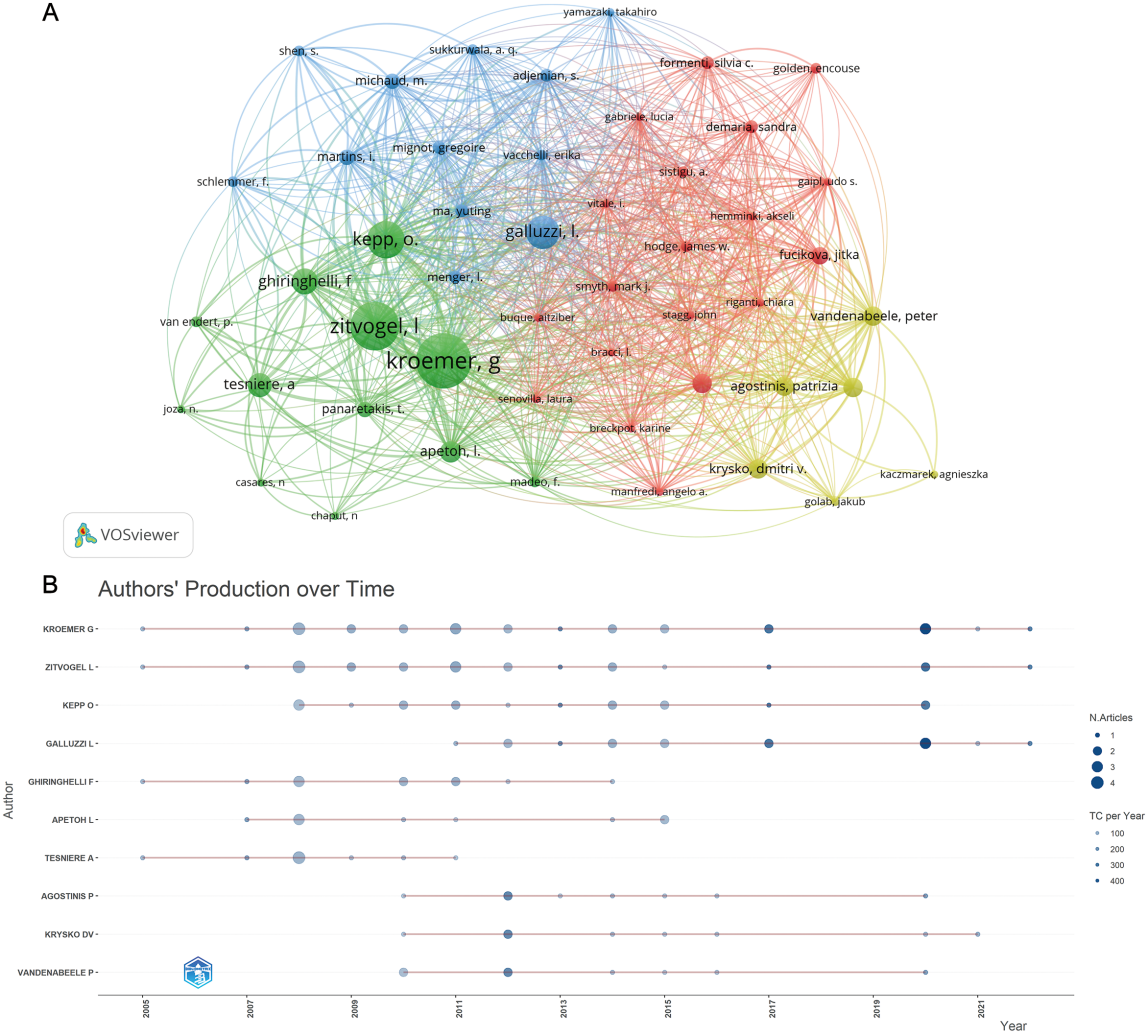
Authors’ analysis of ICD. **(A)** Author co-authorship network. **(B)** Authors’ production over time among leading contributors.

Author-level mapping (**Figure 5**, **Table 5**) identified a core group of prolific and highly cited researchers who have shaped ICD development. Guido Kroemer ranked first with 27 Top 100 papers and 22,173 WoS citations, followed by Laurence Zitvogel with 23 papers and 20,435 WoS citations. Olivier Kepp (17 papers; 13,324 WoS citations) and Lorenzo Galluzzi (15 papers; 13,223 WoS citations) formed a secondary core with substantial output and citation influence. The earliest publication years (PY_start) for Kroemer and Zitvogel were 2005, indicating that they contributed at the field’s inception. Author-level counts reflect participation in the selected top-cited publications rather than fractionalized measures of independent authorship impact.

**Table 5:** Top 10 authors of the top 100 most-cited articles on immunogenic cell death.

Authors	Count	TC (WoS)	Country	PY_start
Kroemer, G	27	22173	FRANCE	2005
Zitvogel, L	23	20435	FRANCE	2005
Kepp, O	17	13324	FRANCE	2008
Galluzzi, L	15	13223	FRANCE	2011
Ghiringhelli, F	11	8786	FRANCE	2005
Tesniere, A	10	7677	FRANCE	2005
Apetoh, L	9	6478	FRANCE	2007
Vandenabeele, P	8	6265	BELGIUM	2010
Agostinis, P	8	6208	BELGIUM	2010
Garg, A	8	6208	BELGIUM	2010

PY, publication Year.

### 3.6 Keyword co-occurrence structure

**Figure 6:**
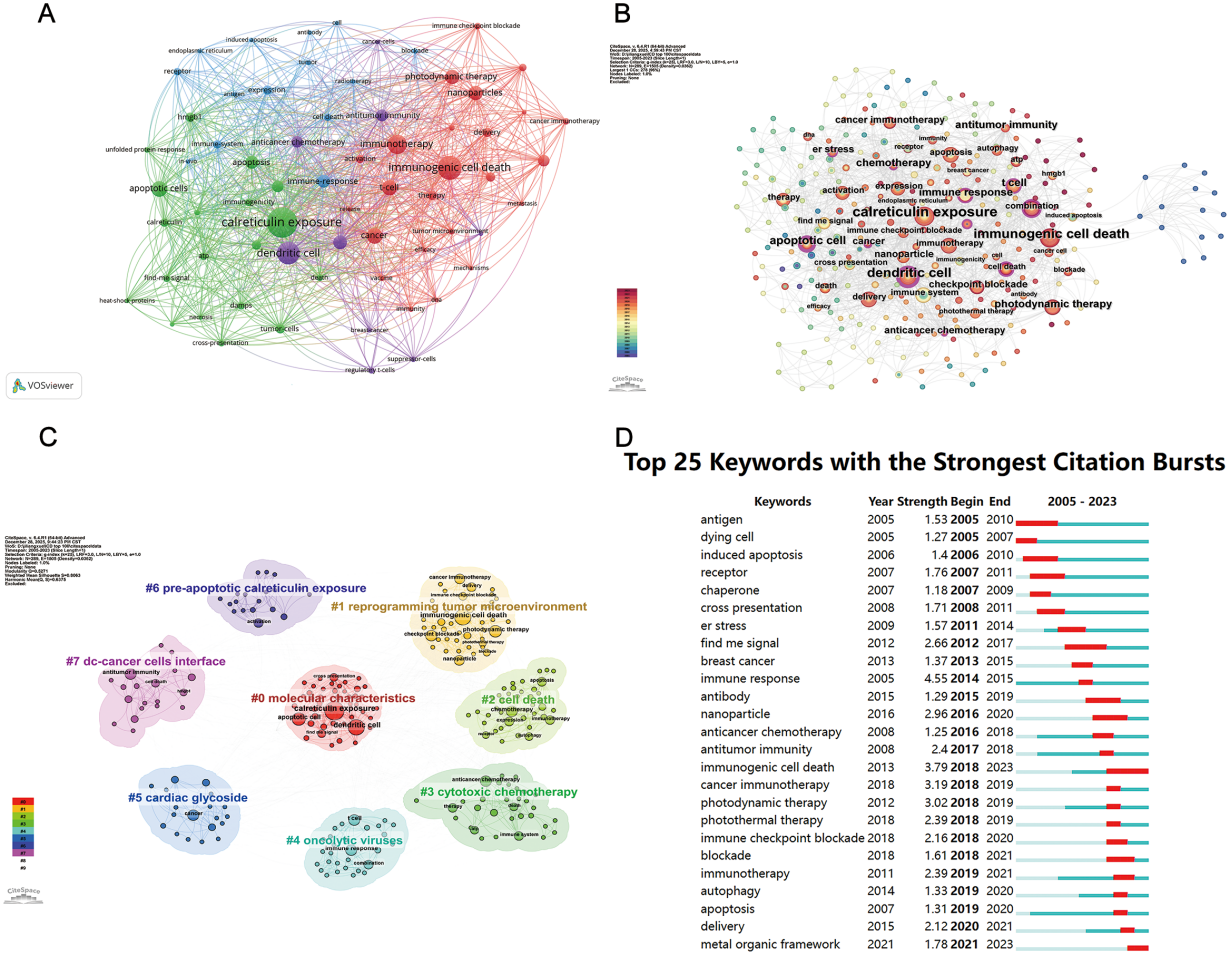
Keyword co-occurrence analysis of ICD. **(A)** Keyword co-occurrence network showing the major conceptual connections among ICD-related terms. **(B)** Keyword density visualization highlighting frequently occurring terms. **(C)** Keyword clustering map showing major thematic groups in the highly cited ICD literature. **(D)** Top 25 keywords with the strongest citation bursts over time, indicating periods of increased research attention.

As shown in **Figures 6A, B**, the keyword network is organized around a set of core terms that correspond to canonical ICD features and immune mechanisms, including immunogenic cell death, calreticulin exposure, dendritic cell, and immunotherapy. Clustering analysis (**Figure 6C**) grouped the keyword landscape into eight major thematic clusters, reflecting the multidisciplinary expansion of ICD research. Cluster #0 (“molecular characteristics”) captures mechanistic features and signaling pathways that define immunogenicity at the cellular level. Cluster #2 (“cell death”) and cluster #6 (“pre-apoptotic calreticulin exposure”) emphasize canonical cell death modalities and hallmark signals. Cluster #3 (“cytotoxic chemotherapy”) aggregates work linking classical chemotherapeutics to immune activation via ICD. Cluster #1 (“reprogramming tumor microenvironment”) reflects research that connects ICD induction to changes in the tumor microenvironment and downstream responsiveness to immunotherapy. Cluster #4 (“oncolytic viruses”) highlights viral platforms as ICD inducers and immunogenic stimulators. Cluster #5 (“cardiac glycoside”) suggests a focused subtopic around repurposed compounds and pharmacologic triggers. Finally, cluster #7 (“DC cancer cell interface”) underscores antigen uptake, cross presentation, and the cellular interactions required to translate tumor cell stress/death into adaptive immunity.

CiteSpace burst detection further identified terms that received rapidly increasing attention during specific time windows, thereby reflecting research frontiers and stage-specific shifts. The Top 25 burst keywords are displayed in **Figure 6D**. Early bursts (2005–2011) were dominated by immune recognition and antigen presentation concepts, including “antigen”, “dying cell”, “induced apoptosis”, “receptor”, “chaperone”, and “cross presentation”. Mid-stage bursts highlighted stress biology and trafficking cues that link intracellular damage responses to immune activation. “ER stress” displayed a burst from 2011 to 2014. “Find me signal” and “breast cancer” indicated increasing attention to recruitment cues and tumor context applications. As the field moved into the late 2010s, bursts increasingly reflected translational integration with immunotherapy and engineered delivery platforms. Notably, “nanoparticle” and “delivery” indicated the growing importance of nanomedicine enabled ICD induction and spatiotemporally controlled immunomodulation. Therapy-related bursts such as “immune checkpoint blockade”, “blockade”, and “immunotherapy” indicate that ICD was increasingly studied in the context of combination immune-oncology strategies. Additionally, modality-specific terms including “photodynamic therapy” and “photothermal therapy” suggest an expanding focus on physical energy-based triggers.

### 3.7 Topic evolution across time

**Figure 7:**
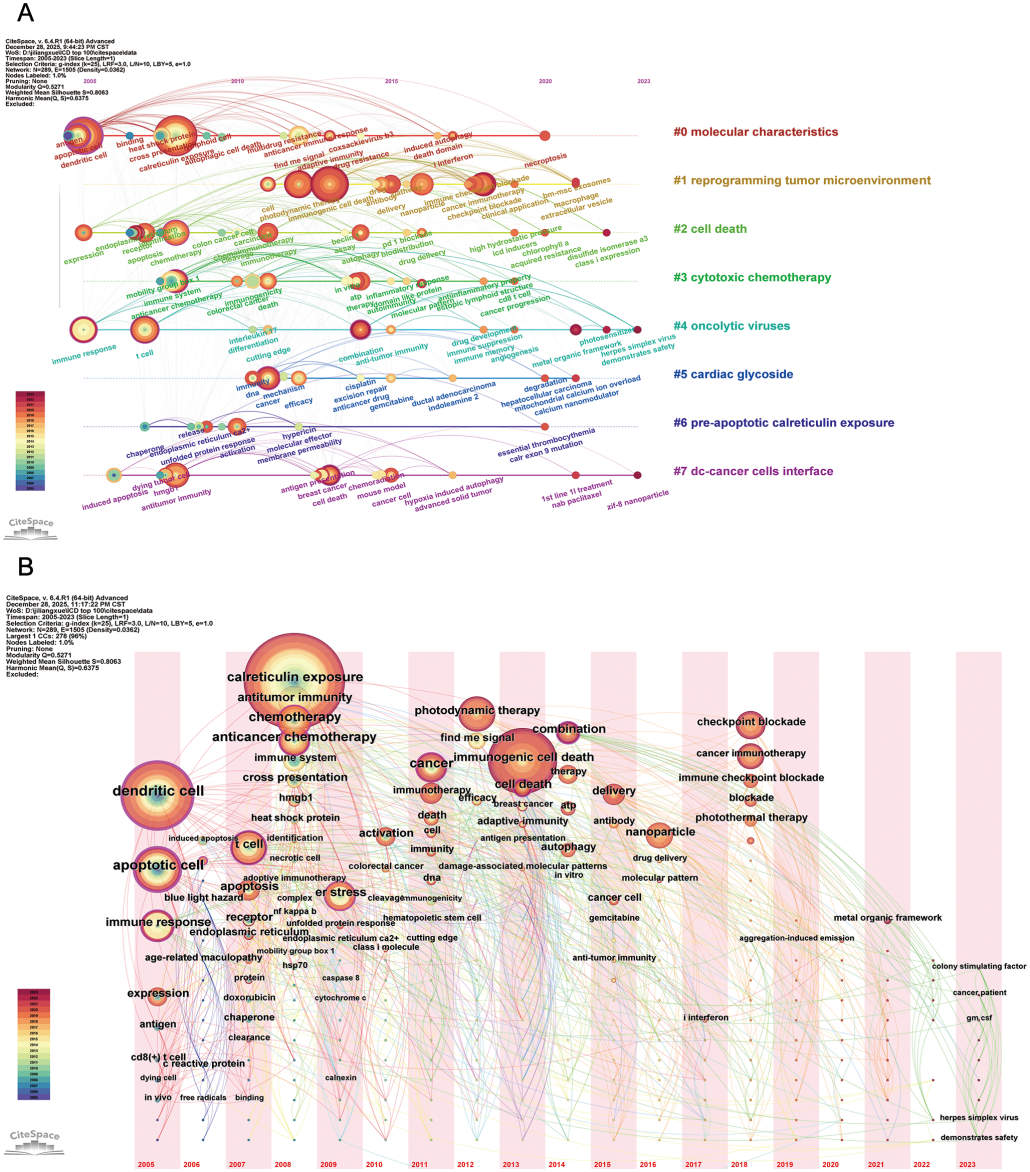
Evolution of ICD research topics over time based on keyword timeline analysis. **(A)** Keyword timeline view showing the persistence and transition of major thematic clusters across the study period. **(B)** Time-zone view showing the chronological emergence of representative keywords, from early mechanistic themes related to immunogenic cell death and antigen presentation to later topics involving immunotherapy, delivery systems, phototherapy, and nanomedicine.

**Figure 7** summarizes thematic evolution using two complementary visualizations: a keyword timeline view and a time zone view. The timeline view demonstrates that early ICD research concentrated on the interface between dying tumor cells and APC, with themes such as dendritic cells, cross presentation, and apoptosis-related immunogenicity appearing prominently in the earlier slices. Over time, mechanistic themes related to calreticulin exposure and ER stress persisted and interconnected with broader immunotherapy themes. In the mid to late period, terms associated with chemotherapy and radiotherapy-based induction gained prominence, consistent with the consolidation of ICD as a mechanism that partly explains immunostimulatory effects of conventional treatment modalities.

The time zone view highlights a shift toward integrative and engineering-enabled strategies in recent years. Themes related to immune checkpoint blockade and cancer immunotherapy appear later and connect to multiple earlier clusters, suggesting that ICD operates as a bridging mechanism that links cell death induction to adaptive immune engagement and to responsiveness to checkpoint inhibition. Meanwhile, recent nodes associated with nanomedicine, delivery, phototherapy, and metal–organic framework platforms occupy the most recent time slices, reflecting an expanding toolkit for ICD induction and a move toward controllable, translationally oriented approaches. Overall, the keyword timeline and time-zone views visually supported a shift in thematic emphasis within the top-cited ICD literature, from early mechanistic and immune-recognition topics toward later interdisciplinary themes involving immunotherapy combinations, engineered delivery platforms, and physical energy-based treatment modalities.

## 4 Discussion

### 4.1 Principal findings

This bibliometric analysis of the 100 most-cited ICD publications provides a focused overview of the field’s intellectual structure and thematic evolution. The included papers were published between 2005 and 2023, with a larger proportion appearing after 2011 and the highest number published in 2019 within this selected dataset. Importantly, this temporal pattern should be interpreted as the publication-year distribution of papers that later accumulated high citation impact, rather than as a measure of overall annual productivity in the ICD field. Such a distribution is inherently influenced by citation accrual time, publication age, and article type; therefore, older landmark studies and review articles may be more likely to appear in a top-cited dataset, whereas more recent studies may be underrepresented. The inclusion of both original articles and reviews further suggests that citation visibility in ICD research has been driven by primary mechanistic discoveries as well as conceptual synthesis (2, 28).

The most cited publications highlight the foundational role of early mechanistic work in defining ICD. The top ranked articles focused on ICD in cancer therapy, calreticulin exposure, and DAMP-associated signaling (29, 31). These publications helped establish calreticulin exposure, ATP secretion, HMGB1 release, antigen uptake, DC activation, and cross presentation as key concepts in the field (32, 33). Thus, ICD should not be regarded merely as a collection of dying cell markers. It provides a biological framework for understanding when tumor destruction may support adaptive antitumor immunity (30).

### 4.2 Global landscape and disciplinary expansion

The country and institution analyses suggest that highly cited ICD research was concentrated within several closely connected research networks. The United States contributed the largest number of top-cited publications, whereas France had the highest total citation count. Several French institutions, including Université Paris, Gustave Roussy, INSERM, Hôpital Européen Georges Pompidou, and Centre de Recherche des Cordeliers, occupied prominent positions among the leading institutions. This pattern is consistent with the important role of French research groups in shaping ICD terminology, experimental standards, and mechanistic models (34, 35). However, these institutional rankings should be interpreted with caution, as multiple leading French institutions may reflect overlapping affiliations of the same collaborative author networks. Belgium showed high betweenness centrality in the country collaboration network, suggesting a bridging role in international knowledge flow. China also ranked among the leading countries in publication count and citation totals, indicating its growing contribution to highly visible ICD research.

The author analysis further supports the presence of a cohesive intellectual core. Guido Kroemer, Laurence Zitvogel, Olivier Kepp, and Lorenzo Galluzzi ranked among the most productive and most cited authors in the top 100 set. Their early publication years and high citation totals suggest sustained involvement from the formative stage of ICD research to later conceptual expansion. This author-level concentration helps explain why ICD terminology, DAMPs-based validation strategies, and mechanistic expectations became relatively standardized across highly cited publications.

The journal distribution reflects the broadening disciplinary scope of ICD research. Nature Communications published the largest number of top 100 papers, while Advanced Materials, Cell Death & Differentiation, ACS Nano, Journal of the American Chemical Society, and Oncogene also contributed multiple highly cited papers. The appearance of materials science and nanomedicine journals among the leading sources suggests that ICD has increasingly been used as a therapeutic design principle rather than only as a biological observation (36). Nanoparticles, metal-organic frameworks, photosensitizers, sonodynamic platforms, and tumor-responsive delivery systems are being developed to coordinate antigen release, danger signaling, innate immune sensing, DC recruitment, and T cell priming (37, 38). Nevertheless, high citation visibility should not be equated with translational maturity. Future studies should distinguish platforms that truly induce immune-dependent tumor control from those that mainly increase DAMPs or produce short-term tumor inhibition (39).

### 4.3 Thematic evolution and a hypothesis-generating four-bottleneck framework

The keyword co-occurrence and burst analyses showed a clear thematic transition. Early burst terms were mainly related to antigen recognition, dying cells, chaperones, cross presentation, and endoplasmic reticulum stress. Later terms increasingly included immune checkpoint blockade, immunotherapy, nanoparticles, delivery, photodynamic therapy, and photothermal therapy. These findings are consistent with the journal distribution, in which both immunology journals and materials-oriented journals were represented among the leading sources. Together, the keyword and journal patterns suggest that ICD research has moved from mechanistic definition toward combination immune-oncology and controllable treatment engineering (40).

Based on these bibliometric patterns and the broader ICD literature, we propose a hypothesis-generating four-bottleneck framework to organize future ICD research. This framework should be understood as a conceptual synthesis rather than a direct output of the keyword co-occurrence or burst analyses. It is not intended to indicate that ICD-based strategies have reached clinical readiness. The first bottleneck is the quality of tumor cell stress and death, including the timing and magnitude of calreticulin exposure, ATP secretion, HMGB1 release, type I interferon signaling, antigen availability, and inflammatory context (41, 42). The second is APC competence, particularly dendritic cell uptake, maturation, and cross-presentation (43). The third is tumor microenvironment permissiveness, which may be limited by myeloid suppression, hypoxia, adenosine metabolism, inhibitory cytokines, macrophage polarization, and impaired T-cell trafficking (44, 45). The fourth is therapeutic synchronization, in which dose, timing, and sequence determine whether cytotoxic therapy, local therapy, delivery systems, and checkpoint blockade can cooperate (46).

This proposed framework may serve as a conceptual tool for organizing experimental questions raised by the observed bibliometric trends. For example, the increasing attention to nanoparticles and delivery systems may reflect attempts to improve local immune activation and treatment coordination (47). The burst of immune checkpoint blockade and immunotherapy indicates growing interest in combining ICD induction with T-cell reinvigoration (48). Meanwhile, persistent terms such as calreticulin exposure, ER stress, dendritic cells, and cross-presentation suggest that mechanistic validation remains central to ICD research (49). However, these bibliometric signals should not be interpreted as proof of clinical readiness. Future ICD studies should therefore move beyond demonstrating DAMP induction and clarify which immunological bottleneck is being addressed through functional validation and clinically relevant models.

### 4.4 Translational implications

The translational implications discussed here should be interpreted as hypothesis-generating considerations rather than conclusions of clinical readiness derived from bibliometric data. Bibliometric trends can identify areas of increasing research attention, such as immunotherapy combinations, engineered delivery platforms, and physical energy-based therapies, but they cannot determine whether these strategies are clinically effective. Therefore, marker-based evidence, such as increased calreticulin exposure, ATP release, or HMGB1 secretion, should be viewed as preliminary rather than definitive (50). Stronger evidence should include time-resolved DAMPs, antigen uptake by APC, DC maturation, cross presentation, antigen-specific CD8+T cell activation, pathway dependence experiments, and validation in immunocompetent tumor models (51). The most convincing evidence should demonstrate that antitumor efficacy depends on adaptive immunity, for example through vaccination or rechallenge experiments, immune cell depletion, blockade of key danger signal pathways, or loss of function and rescue approaches (52).

For clinicians and translational researchers, ICD may be better understood as a treatment context modifier rather than an independent endpoint (53). The presence of ICD hallmarks may increase the likelihood that a therapy supports antitumor immunity, but clinical benefit also depends on baseline immune context, antigen presentation capacity, tumor microenvironment status, and combination design (54). This interpretation is consistent with the observed shift toward immune checkpoint blockade, immunotherapy, delivery systems, and phototherapy in the later keyword bursts. Tumors with preserved DC function, intact antigen presentation machinery, and reversible immune suppression may be more likely to benefit from ICD-inducing combinations. In contrast, tumors with severe DC exclusion, defective antigen presentation, or dominant suppressive myeloid circuits may require additional microenvironmental reprogramming before ICD induction can generate durable immunity (55).

The close connection between ICD and immune checkpoint blockade further supports this view (56). ICD-inducing treatments can promote antigen release and immune priming, whereas checkpoint inhibitors mainly relieve inhibitory signaling during T cell activation and effector function (57). These mechanisms are complementary, but synergy is not guaranteed. Poorly timed cytotoxic therapy may impair immune cells or promote suppressive repair programs. Therefore, clinical trials of ICD-based strategies should incorporate rational sequencing, immune monitoring, and patient selection (58). ICD markers alone are unlikely to be sufficient predictors of therapeutic benefit, and future translational studies should prioritize functional immune evidence and clinically relevant validation over marker-based claims alone.

### 4.5 Limitations and future directions

Several limitations should be acknowledged. First, the high-specificity search strategy using the terms “immunogenic cell death” and “cell death, immunogenic” may also have missed relevant studies that addressed DAMPs signaling, antigen presentation, or therapy-induced antitumor immunity without explicitly using ICD-related terminology. Second, the top-cited design inevitably favors older publications, reviews, and studies from well-established research groups, while recent, lower-cited, negative, or emerging studies may be underrepresented. The annual distribution should be interpreted as the publication-year distribution of included top-cited papers, not as overall field productivity. Third, country/region, institution, and author analyses were based on full counting, so collaborative papers could be counted repeatedly across affiliations; these rankings reflect affiliation-based participation and collaborative visibility rather than independent or fractionalized impact. Finally, keyword co-occurrence, clustering, timeline, and burst analyses reveal research attention and thematic associations, but they cannot establish causality, therapeutic efficacy, clinical value, or clinical readiness. The proposed four-bottleneck framework should be regarded as a conceptual and hypothesis-generating model, and future ICD research should prioritize functional validation, immune-context-aware biomarkers, clinically relevant models, and rational combination strategies.

The next stage of ICD research should move beyond identifying additional ICD inducers and toward building predictive, function-based, and clinically testable models (59). Integrated biomarkers are needed to combine tumor cell stress signals with immune context variables, such as DC abundance and activation, antigen presentation capacity, T cell infiltration, myeloid suppression, ectonucleotidase activity, and interferon related pathways (60). Preclinical studies should compare different ICD-inducing strategies across immune hot, immune excluded, and immune cold tumor models (61). Clinical studies should include longitudinal tissue and blood sampling to determine whether ICD-based regimens expand antigen-specific T cells, broaden antigen recognition, reverse immune suppression, and generate durable memory.

## 5 Conclusion

In conclusion, this study integrates bibliometric and content analyses of the 100 most-cited publications on ICD over the past two decades, showing that the field has progressed from establishing the ICD concept and hallmark DAMPs signals to deeper mechanistic work and, more recently, strong translational integration with immunotherapy, nanomedicine-enabled delivery, and phototherapy-based induction strategies. Although ICD has become a critical bridge connecting tumor killing with adaptive immune priming, future efforts should prioritize functional immunological evidence over merely increased biomarkers. It is essential to develop biomarkers more closely aligned with the tumor microenvironment and to design more rational combination therapies, thereby advancing ICD toward reproducible and translatable clinical benefits.

## Data Availability

The original contributions presented in the study are included in the article/**Supplementary Material**, further inquiries can be directed to the corresponding author/s.
